# Increasing Maternal Age Is Associated with Taller Stature and Reduced Abdominal Fat in Their Children

**DOI:** 10.1371/journal.pone.0058869

**Published:** 2013-03-20

**Authors:** Tim Savage, José G. B. Derraik, Harriet L. Miles, Fran Mouat, Paul L. Hofman, Wayne S. Cutfield

**Affiliations:** 1 Liggins Institute, University of Auckland, Auckland, New Zealand; 2 Gravida: National Centre for Growth and Development, Auckland, New Zealand; 3 Starship Children's Hospital, Auckland District Health Board, Auckland, New Zealand; INRA, France

## Abstract

**Background:**

Maternal age at childbirth continues to increase worldwide. We aimed to assess whether increasing maternal age is associated with changes in childhood height, body composition, and metabolism.

**Methods:**

277 healthy pre-pubertal children, born 37–41 weeks gestation were studied. Assessments included: height and weight corrected for parental measurements, DEXA-derived body composition, fasting lipids, glucose, insulin, and hormonal profiles. Subjects were separated according to maternal age at childbirth: <30, 30–35, and >35 years.

**Results:**

Our cohort consisted of 126 girls and 151 boys, aged 7.4±2.2 years (range 3–10); maternal age at childbirth was 33.3±4.7 years (range 19–44). Children of mothers aged >35 and 30–35 years at childbirth were taller than children of mothers aged <30 years by 0.26 (p = 0.002) and 0.23 (p = 0.042) SDS, respectively. There was a reduction in childhood BMISDS with increasing maternal age at childbirth, and children of mothers aged >35 years at childbirth were 0.61 SDS slimmer than those of mothers <30 years (p = 0.049). Children of mothers aged 30–35 (p = 0.022) and >35 (p = 0.036) years at childbirth had abdominal adiposity reduced by 10% and 13%, respectively, compared to those in the <30 group. Children of mothers aged 30–35 years at childbirth displayed a 19% increase in IGF-I concentrations compared to offspring in <30 group (p = 0.042). Conversely, IGF-II concentrations were lower among the children born to mothers aged 30–35 (6.5%; p = 0.004) and >35 (8.1%; p = 0.005) compared to those of mothers aged <30 years. Girls of mothers aged 30–35 years at childbirth also displayed improved HOMA-IR insulin sensitivity (p = 0.010) compared to girls born to mothers aged <30 years.

**Conclusions:**

Increasing maternal age at childbirth is associated with a more favourable phenotype (taller stature and reduced abdominal fat) in their children, as well as improved insulin sensitivity in girls.

## Introduction

Over the past three decades, maternal age at first childbirth has increased by approximately 4 years in most developed countries [1]. This shift in reproductive behaviour means that most children are currently born to mothers aged over 30 years [1]. The reasons for the postponement of childbirth include increased availability of contraception, greater educational and career opportunities for women, economic pressures, and personal choice [2].

There is growing interest in the possible contribution of maternal age at childbirth to the future health of the offspring. Increasing maternal age at childbirth has been linked to adverse health effects on their children, including greater risk of type 1 diabetes [3], as well as higher blood pressure in childhood [4] and higher rates of type 2 diabetes [5] in adulthood. Increasing maternal age is also associated with increased rates of obstetric and perinatal complications, including fetal loss, pre-eclampsia, premature delivery, and low birth weight [6].

Older mothers also have children with increased rates of chromosomal and other genetic disorders, which are thought to be largely due to a decline in oocyte quality [7]. The timing of the onset and rate of this decline in oocyte quantity and quality is a subject of on-going debate [7]. Nonetheless, there is a substantial reduction in female fertility from 30 years of age, which may signal the start of oocyte decline [8]. However, some authors suggest that oocyte quality and quantity declines most sharply after 35 years of age [9], while others describe a gradual decline from menarche to menopause [10]. Increasing age is also associated with physiological changes in the mother's reproductive system, such as alterations in gonadotropin and other hormone levels [11], [12].

These alterations in the early fetal environment may contribute to changes in the physical characteristics and disease risk of the offspring, but it is unknown whether maternal age directly affects the growth or metabolism of their children. Thus, in this study we aimed to assess whether increasing maternal age would be associated with changes in height, body composition, as well as lipid and metabolic profiles in the offspring in childhood.

## Methods

### Ethics statement

Ethics approval for this study was provided by the Northern Y Regional Ethics Committee (Ministry of Health, New Zealand). Written informed consent was obtained from parents or guardians, as well as verbal or written consent from each child as was appropriate to their age.

### Subjects

**Figure 1:**
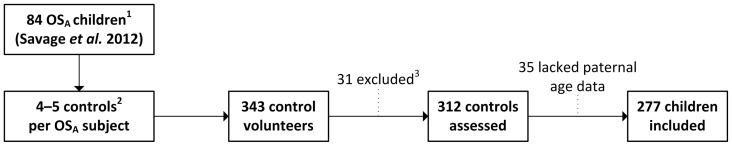
Summary of the study's recruitment process. ^1^ OS_A_ children had been conceived via ovarian stimulation, and were examined in Savage *et al.* [13]. ^2^ Controls were friends of OS_A_ children to ensure similar age group, ethnicity, and socio-economic status. ^3^ 22 children were born small-for-gestational age and/or premature; 5 were pubertal; 3 were born to a mother with gestational diabetes/glucose intolerance; and one child was on medication known to influence growth.

We undertook a large project examining the effects of parental and prenatal factors in the offspring. From this larger project, we have examined the impact of conception with ovarian stimulation drugs on the growth and metabolism of children [13]. Children conceived after ovarian stimulation were asked to invite family friends and school friends who were naturally conceived to participate in the study as controls [13], so that these controls were recruited randomly by study participants. Thus, in this current study we assessed the entire naturally conceived cohort that was recruited from this larger project (Figure 1).

Only healthy, developmentally normal, pre-pubertal children aged 3–10 years, born 37–41 weeks gestation were studied. All children were of New Zealand European ethnicity, naturally conceived, born to singleton pregnancies, and of birth weight appropriate-for-gestational-age (birthweight >-2 and <2 standard deviation scores (SDS)). Exclusion criteria also included signs of puberty (Tanner stage 2 breast development in girls and testicular volume >3 ml in boys or evidence of adrenarche), receiving medication that could affect insulin sensitivity or growth, and having a first degree relative with diabetes. Children were excluded if born to mothers with gestational diabetes, pre-eclampsia, gestational or pre-existing hypertension, chronic illnesses, or maternal drug use (including tobacco and alcohol) during pregnancy. All participants were of higher socio-economic status according to their residential address and the ‘decile score’ of the school they attended [14]. A decile score reflects the socio-economic status of school communities, where decile 1 indicates lowest and 10 highest socio-economic status [14]. The ‘decile score’ is a comprehensive assessment of community affluence, which takes into account a number of factors, such as household income, parental occupation and educational qualifications, number of occupants per dwelling size, and government welfare benefits [15], [16]. All participants in the study were from schools of decile 9 or 10.

### Study design

All clinical assessments were carried out by a single researcher at the Maurice & Agnes Paykel Clinical Research Unit (Liggins Institute, University of Auckland). Standing and sitting height were measured using a Harpenden stadiometer. Children's weight and body composition were assessed using dual-energy X-ray absorptiometry (DEXA Lunar Prodigy 2000; General Electric, Madison, WI, USA). Apart from total body fat percentage, the DEXA-derived parameter of interest was abdominal adiposity. The latter was expressed as the android fat to gynoid fat ratio and provided by the manufacturer's software based on an automated sectioning of specific areas of the body [17]. A number of studies in children have shown that proportionally greater adiposity in the upper body (i.e. android or male fat) is associated with adverse metabolic outcomes [18], [19]. Each child also had a bone age X-ray to assess biological maturity, which was blindly assessed by a single paediatric endocrinologist using pre-established standards [20].

Children's birth weight, height, body mass index (BMI), and parental height were transformed into SDS [21], [22], [23]. Maternal and paternal heights, weights, and BMI were recorded. Mid-parental height SDS (MPHSDS) was calculated for each child [24]. Children's height SDS were then individually corrected for their genetic potential (parents' heights) using the formula: child's height SDS minus MPHSDS. Parents' BMI were transformed into SDS, and the mean parental BMISDS (MPBMISDS) was calculated for each child [25]. Maternal obstetric history was also recorded to clarify parity and relevant medical history.

Following an overnight fast, blood samples were drawn from each child for assessment of total cholesterol, high-density lipoprotein cholesterol (HDL-C), low-density lipoproteins cholesterol (LDL-C), triglycerides, insulin-like growth factor 1 (IGF-I), IGF-II, IGF binding protein 3 (IGFBP-3). Children also had glucose and insulin levels measured, and insulin sensitivity evaluated using the homeostasis model assessment of insulin resistance (HOMA-IR) [26].

Plasma insulin was measured using an Abbott AxSYM system (Abbott Laboratories, Abbott Park, IL, USA) by microparticle enzyme immunoassay (Abbott Diagnostics, Wiesbaden, Germany) with an inter-assay coefficient of variation (CV) of <5%. Glucose, triglycerides, total cholesterol, HDL-C, and LDL-C concentrations were measured on a Hitachi 902 autoanalyser (Hitachi High Technologies Corporation, Tokyo, Japan) by enzymatic colorimetric assay (Roche, Mannheim, Germany) with an inter-assay CV of 1.2% for glucose, and <5% for total cholesterol, triglycerides, HDL-C, and LDL-C. Commercially available ELISAs (R&D Systems, Minneapolis, MN, USA) were used to measure plasma IGF-I (DSL-100, intra-assay CV 2.8%, inter-assay CV 9.2%), IGFBP-3 (DSL-10-6600, intra-assay CV 3.1%, inter-assay CV 9.9%), and IGF-II (Meddiagnost, Reutlingen Germany; E-30, intra-assay CV 1.9%, inter-assay CV 6.3%).

### Statistical analysis

To examine the possible non-linear effects of maternal age on measured outcomes, subjects were separated according to maternal age at childbirth using two maternal age thresholds: 30 [8] and 35 years [9]. Subjects were divided into 3 groups: children born to mothers aged less than 30 years of age (<30), 30 to 35 years (30–35), or greater than or equal to 35 years (>35).

Demographics of the study cohort were presented as means ± standard deviation, and compared between groups using one-way ANOVA. Random effect mixed models were used to compare outcomes among maternal age groups, and accounted for important confounding factors including gender, birth weight SDS, gestational age, birth order, and paternal age. The maternal identification number was considered as a random factor to account for the clustering of siblings. Other factors were controlled for as required, depending on the outcome response of interest: for lipids, hormones, and outcomes associated with glucose homeostasis – age and BMISDS were included; for anthropometric data – the appropriate parental factor (i.e. MPBMISDS or MPHSDS). The differences between groups were estimated and tested using pairwise comparisons. The interaction effect between group and gender was tested in all models. Outcomes were only assessed separately for boys and girls when there was an indication of a differential response to maternal age between genders. Response variables on glucose homeostasis were log-transformed to approximate normality. Statistical analyses were performed using SAS version 9.2 (SAS Institute Inc. Cary NC, USA). All statistical tests were two-sided and maintained at a 5% significance level. Outcome data were presented as estimated marginal means with associated 95% confidence intervals.

## Results

**Figure 2:**
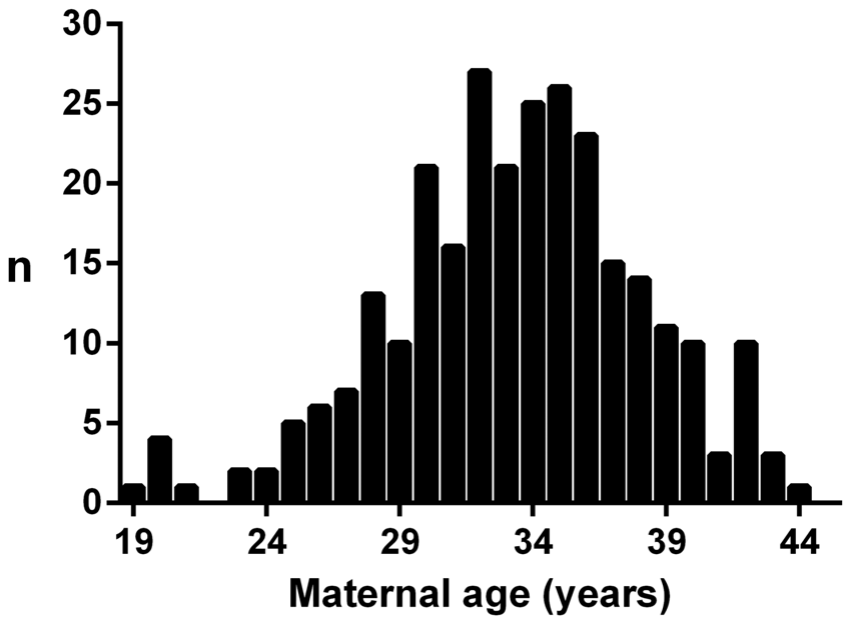
Distribution of maternal age at childbirth in the study cohort.

A total of 343 children volunteered to participate, but 31 were excluded: 22 children were born small-for-gestational age and/or premature, 5 were pubertal, 3 were born to a mother with gestational diabetes/glucose intolerance, and one child was on medication known to influence growth (Figure 1). Of the remaining 312 controls, a further 35 had to be excluded due to incomplete paternal age data (Figure 1). Thus, our study cohort consisted of 277 children (126 girls and 151 boys) aged 3–10 years (7.4±2.2). The offspring of 196 mothers were included in this study, as there were 71 sibling groups of 2 or 3 children. Maternal age at the time of childbirth ranged from 19–44 years (33.3±4.7 years) (Figure 2), and this maternal age distribution is representative of New Zealand European families of higher socio-economic status [27].

Age, sex ratio, birth weight SDS, and gestational age were similar among groups (Table 1). There were no differences in maternal BMI, mean parental BMI, or duration and rate of breast feeding among groups (data not shown). In addition, children in all maternal age groups had similar biological maturity as assessed by bone age X-rays (all p>0.79; Table 1).

**Table 1:** Demographics of the study cohort according to their mother's age at childbirth.

	Maternal age at childbirth		
	<30 years	30–35 years	>35 years
n	62	103	112
Sex ratio (% boys)	55	58	50
Age (years)	7.6±2.3	7.6±2.0	7.0±2.2
Bone age – chronological age (years)	−0.21±0.97	−0.10±0.80	−0.19±0.71
Gestational age (weeks)	39.7±1.2	39.8±1.2	39.5±1.2
Birth weight SDS	0.19±0.98	0.28±1.02	0.12±0.86

Data are means ± standard deviations.

### Anthropometry

**Figure 3:**
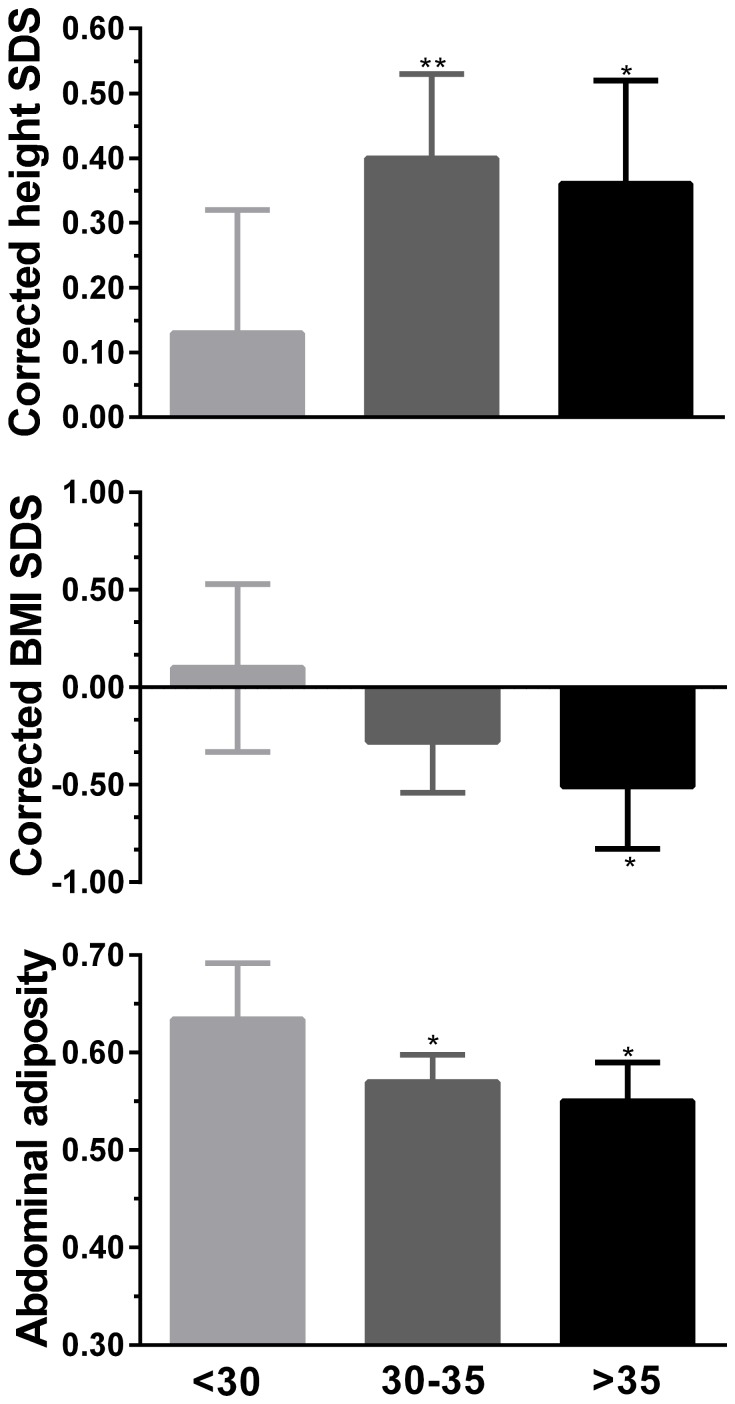
Height and BMI SDS corrected for mean parental height and mid-parental BMI SDS, respectively, among the offspring of mothers of different ages at childbirth. Data are estimated marginal means and 95% confidence intervals, adjusted for other confounding factors in multivariate models (including paternal age). *p<0.05 and **p<0.01 vs children of mothers aged <30 years at childbirth. Abdominal adiposity is represented by the android fat to gynoid fat ratio. The 95% confidence intervals for the differences between groups are provided in Table S1.

When corrected for their genetic potential, children of mothers aged 30–35 and >35 years were 0.26 (p = 0.002) and 0.23 (p = 0.042) SDS taller than children of mothers aged <30 (Figure 3; Table S1). There was a reduction in childhood BMI SDS with increasing maternal age at childbirth, so that children born to mothers aged >35 years were 0.61 SDS slimmer than those of mothers <30 years (p = 0.049) (Figure 3; Table S1). Increasing maternal age at childbirth was also associated with improved fat distribution in the offspring, so that children in both 30–35 (p = 0.022) and >35 groups (p = 0.036) had abdominal adiposity that was 10% and 13% lower, respectively, compared to children of mothers aged <30 years (Figure 3; Table S1).

A leaner phenotype was more apparent among boys born to mothers aged 30–35 and >35 years. In this group, increasing maternal age at childbirth was associated with a decrease in total body fat percentage, which was 12.9% in boys born to mothers aged >35 years compared to 14.7% (p = 0.053) and 16.6% (p = 0.015) in the boys of mothers aged 30–35 and <30 years at childbirth, respectively. Among girls, total body fat percentage was not different among groups (data not shown).

### Metabolic and hormonal profiles

Children of mothers aged 30–35 years at childbirth displayed a 19% increase in IGF-I concentrations compared to the offspring of mothers aged <30 years (p = 0.042; Table 2; Table S1). Conversely, IGF-II concentrations were lower among the children of mothers aged 30–35 (6.5%; p = 0.004) and >35 (8.1%; p = 0.005) compared to those of the mothers aged <30 years at childbirth (Table 2; Table S1). There were no observed effects of maternal age at childbirth on their children's lipid profiles (Table 2; Table S1).

**Table 2:** Lipid and IGF profiles in childhood according to maternal age at childbirth.

	Maternal age at childbirth		
	<30 years	30–35 years	>35 years
**n**	62	103	112
**Lipid profile**			
Total cholesterol (mmol/l)	4.35 (4.12–4.51)	4.33 (4.20–4.47)	4.32 (4.15–4.51)
LDL-C (mmol/l)	2.52 (2.31–2.74)	2.57 (2.45–2.69)	2.51 (2.35–2.67)
HDL-C (mmol/l)	1.41 (1.31–1.52)	1.34 (1.28–1.40)	1.37 (1.29–1.45)
LDL-C : HDL-C	1.77 (1.58–2.00)	1.89 (1.77–2.02)	1.83 (1.68– 2.00)
**Hormones**			
IGF-I (µg/l)	97 (81–113)	115 (106–125)*	111 (99–123)
IGF-II (µg/l)	790 (759–823)	739 (721–757)**	726 (702–749)**
IGFBP-3 (ng/ml)	2803 (2501–3104)	2682 (2508–2855)	2596 (2373–2818)

Data are estimated marginal means and 95% confidence intervals adjusted for other confounding factors in the multivariate models (including paternal age). *p<0.05 and **p<0.01 vs children of mothers aged <30 years at childbirth. The 95% confidence intervals for the differences between groups are provided in Table S1.

Maternal age at childbirth did not affect parameters of glucose homeostasis among boys. However, girls of mothers aged 30–35 years at childbirth displayed improved insulin sensitivity (p = 0.009) compared to the girls of mothers aged <30, as expressed by lower HOMA-IR values (Table 3; Table S2).

**Table 3:** Parameters of glucose homeostasis among boys and girls according to maternal age at childbirth.

	Maternal age at childbirth		
	<30 years	30–35 years	>35 years
**Boys (n)**	34	65	52
Insulin sensitivity (HOMA-IR)	0.99 (0.79–1.24)	0.98 (0.87–1.11)	0.97 (0.82–1.14)
Fasting glucose (mmol/l)	4.70 (4.54–4.86)	4.80 (4.70–4.90)	4.85 (4.71–4.98)
Fasting insulin (mU/l)	4.79 (3.90–5.88)	4.52 (4.04–5.07)	4.47 (3.84–5.20)
**Girls (n)**	28	47	51
Insulin sensitivity (HOMA-IR)	1.49 (1.14–1.95)	1.00 (0.86–1.17)^**^	1.14 (0.95–1.37)
Fasting glucose (mmol/l)	4.93 (4.74–5.14)	4.70 (4.59–4.81)^*^	4.75 (4.62–4.89)
Fasting insulin (mU/l)	6.75 (5.30–8.61)	4.85 (4.22–5.57)^*^	5.42 (4.86–6.41)

Data are estimated marginal means and 95% confidence intervals adjusted for other confounding factors in the multivariate models (including paternal age). ^*^p<0.05 and ^**^p<0.01 for comparisons with <30 group. The 95% confidence intervals for the ratios between estimated marginal means are provided in Table S2.

## Discussion

Our study shows that increasing maternal age at childbirth is associated with a more favourable phenotype in their children. This includes an increase in height, a reduction in abdominal fat, as well as improved insulin sensitivity in girls.

On average, children of mothers aged 30–35 and >35 years at childbirth were 1.5 cm taller than the offspring of mothers aged less than 30 years. This difference in stature was accompanied by higher serum IGF-I concentrations in the children born to older mothers, who were taller than children born to mothers aged <30 years. This corroborates our findings, as IGF-I is an important mediator of childhood growth [28]. Importantly, our finding of taller stature with increasing maternal age was present after correction for genetic height, the most important determinant of childhood height [24]. Further, we also corrected for other factors known to influence childhood height, including gestational age, birth weight [29], and birth order [30], as well as accounting for socio-economic status through our cohort selection process [31]. As children in all maternal age groups had similar biological maturity as assessed by bone age X-rays, it is likely that the observed height differences will persist into adulthood [24].

Increasing maternal age was also associated with a decreased BMI and a reduction in abdominal fat in their children. Increased abdominal fat is a component of the metabolic syndrome in childhood and adulthood [32]. Thus, we suggest that children born to mothers over 30 years of age may be at a lower risk of metabolic disease and obesity compared to those of younger mothers. The observed improvement in insulin sensitivity among girls born to older mothers would support this assertion, as a reduction in insulin sensitivity is predictive of the metabolic syndrome in adulthood [33], [34].

Children born to younger mothers also had higher IGF-II concentrations that may be associated with their increased adiposity, as elevations in serum IGF-II concentrations are associated with increased body fat [35]. Causal factors for childhood obesity have been extensively investigated [36], but previous studies have found no impact of maternal age on childhood BMI [37], [38], [39]. However, in those studies, maternal age was just one of many secondary study outcomes in populations from all socio-economic groups [36]. Since both maternal age at childbirth and obesity risk are strongly associated with socio-economic status [2], [40], previous studies might have been unable to accurately detect subtle effects of maternal age on childhood BMI or body composition.

There are a very limited number of studies examining the physical and metabolic outcomes in the offspring associated with maternal age at childbirth [3], [4], [5]. These studies described subtle increases in blood pressure [4] and type 1 diabetes risk [3] in childhood, and an increase in type 2 diabetes risk in adults [5]. These findings contrast to our observations of a more favourable offspring phenotype in childhood associated with increasing maternal age at childbirth. However, previous studies included either crude adjustments in their analyses for socio-economic status [4] or none at all [3], [5], which is important as lower socio-economic status is known to be associated with increased blood pressure and type 2 diabetes risk [41]. Most importantly, all three studies included subjects who were born small-for-gestational-age, prematurely, and/or of low birth weight [3], [4], [5], groups at a greater risk of developing hypertension and type 2 diabetes [42], [43]. Although Cardwell *et al*. and Lawlor *et al*. adjusted for birth weight and gestational age in their analyses [3], [4], Lammi *et al*. did not [5]. Lower birth weight is associated with higher blood pressure later in childhood and adolescence [44], [45], and birth weight decreases with increasing maternal age at childbirth [6]. Thus, birth weight rather than maternal age may account for Lowler *et al*.′s observations of higher offspring blood pressure in childhood with increasing maternal age at childbirth, as postulated by the authors themselves [4]. However, Cardwell *et al*. did speculate on a number of possible explanations for the increased risk of type 1 diabetes in children born to older mothers, including immunological changes in the mother and subtle genetic changes in the offspring [3]. However, the authors concluded that the mechanisms responsible remain unclear [3].

Similarly, there is no clear single explanation for the observed changes in childhood growth, body composition, and metabolism with increasing maternal age at childbirth in our study. It is possible that pre-natal and/or post-natal environmental factors are responsible for our observations. Increasing maternal age is a well-known risk factor for chromosomal disorders in children [7]. However, it is also likely that more subtle gene alterations, such as epigenetic changes, occur with increasing maternal age. Epigenetic changes are alterations in gene expression not caused by changes in DNA sequence [46], which may lead to alterations in phenotype [47]. Increasing age is associated with an increased frequency of epigenetic modifications in both somatic cells [48] and oocytes [49], [50], [51]. Thus, it is possible that epigenetic changes that occur in maternal oocytes with increasing age are responsible for our findings on childhood growth, body composition, and metabolism.

Increasing maternal age is associated with several physiological changes, including subtle increases in maternal follicle-stimulating hormone (FSH) [52], testosterone and oestrogen [53], [54] levels. Such hormonal changes have been associated with alterations in maternal oocyte DNA, as well as alterations in post-natal growth [55] and metabolism [56] in the offspring. Thus, it is possible that changes in maternal hormones with increasing age alter the *in utero* environment, leading to programmed changes in childhood phenotype.

It is recognised that variations in the post-natal child-rearing environment across the socio-economic spectrum affects childhood growth and body composition [31], [57]. Higher socio-economic status is associated with a taller and slimmer phenotype in childhood; while children reared in lower socio-economic environments tend to be shorter and fatter [31], [58]. Since our cohort was comprised of a homogenous group of children from higher socio-economic families, the child-rearing environment is less likely to explain our findings. The absence of a clear explanation for our findings and those of other studies [3], [4], [5] highlights the need for investigation of the possible mechanisms responsible for changes in phenotype and metabolism in the offspring associated with maternal age at childbirth.

Limitations to our study include a relatively small cohort of 277 healthy pre-pubertal children from approximately 200 mothers. In addition, we studied a homogenous group of children (same ethnicity and higher socio-economic status), which may limit application of our study findings to the general population, particularly to those of lower socio-economic status. However, this homogeneity also meant that we eliminated much of the phenotypic and metabolic variability associated with socio-economic status, thus enabling us to better address the likely effects of maternal age on measured outcomes.

## Conclusion

Our study showed that increasing maternal age at childbirth is associated with taller stature and reduced abdominal fat in their offspring in mid-childhood, as well as improved insulin sensitivity in girls. The triggers and mechanisms responsible for these differences are unclear, but may include a combination of maternal age-related changes in the prenatal and post-natal environment. Our study suggests that the worldwide trend towards increasing maternal age is unlikely to underpin the increase in obesity rates in childhood.

## Supporting Information

Table S1 **Height, body composition, lipid profile, and hormonal profiles in childhood according to maternal age at childbirth.** Data are 95% confidence intervals for the differences between estimated marginal means, adjusted for other confounding factors in the multivariate models (including paternal age). Respective p-values are provided in brackets.
(DOC)

Table S2 **Parameters of glucose homeostasis among boys and girls according to maternal age at childbirth.** Data are 95% confidence intervals for the ratios between estimated marginal means, adjusted for other confounding factors in the multivariate models (including paternal age). Respective p-values are provided in brackets.
(DOC)
