# Supplementary material for: Increasing Maternal Age Is Associated with Taller Stature and Reduced Abdominal Fat in Their Children
Source: PLoS One. 2013 Mar 20;8(3):e58869. doi: 10.1371/journal.pone.0058869 (PMC3604016; doi:10.1371/journal.pone.0058869)
Supplement: Table S1 — Height, body composition, lipid profile, and hormonal profiles in childhood according to maternal age at childbirth. Data are 95% confidence intervals for the differences between estimated marginal means, adjusted for other confounding factors in the multivariate models (including paternal age). Respective p-values are provided in brackets. (DOC) [file pone.0058869.s001.doc]

**Table S1.** Height, body composition, lipid profile, and hormonal profiles in childhood according to maternal age at childbirth. Data are 95% confidence intervals for the differences between estimated marginal means, adjusted for other confounding factors in the multivariate models (including paternal age). Respective p-values are provided in brackets.

|  | **Maternal age at childbirth** | | |
| --- | --- | --- | --- |
|  | **30–35 vs <30** | **30–35 vs >35** | **<30 vs >35** |
| **Corrected height SDS** | 0.10 – 0.42  **(0.002)** | -0.10 – 0.17  (0.64) | -0.45 – -0.01  **(0.042)** |
| **Corrected BMI SDS** | -0.84 – 0.08  (0.10) | -0.17 – 0.62  (0.25) | 0.01 – 1.21  **(0.049)** |
| **Abdominal adiposity** | -0.090 – -0.010  **(0.022)** | -0.165 – 0.163  (0.74) | 0.005 – 0.086  **(0.036)** |
| **Lipid profile** |  |  |  |
| Total cholesterol (mmol/l) | -0.26 – 0.27  (0.99) | -0.22 – 0.23  (0.97) | -0.34 – 0.35  (0.99) |
| LDL-C (mmol/l) | -0.19 – 0.28  (0.69) | -0.14 – 0.26  (0.55) | -0.29 – 0.32  (0.93) |
| HDL-C (mmol/l) | -0.19 – 0.04  (0.21) | -0.12 – 0.07  (0.65) | -0.10 – 0.20  (0.51) |
| LDL-C : HDL-C | -0.09 – 0.43  (0.20) | -0.16 – 0.28  (0.60) | -0.45 – 0.22  (0.51) |
| **Hormones** |  |  |  |
| IGF-I (μg/l) | 0.7 – 36.2  **(0.042)** | -11.1 – 19.2  (0.61) | -37.4 – 8.3  (0.21) |
| IGF-II (μg/l) | -86 – -16  **(0.004)** | -16 – 43  (0.38) | 20 – 109  **(0.005)** |
| IGFBP-3 (ng/ml) | -437 – 195  (0.45) | -177 – 348  (0.52) | -212 – 625  (0.33) |
