# Supplementary material for: Increasing Maternal Age Is Associated with Taller Stature and Reduced Abdominal Fat in Their Children
Source: PLoS One. 2013 Mar 20;8(3):e58869. doi: 10.1371/journal.pone.0058869 (PMC3604016; doi:10.1371/journal.pone.0058869)
Supplement: Table S2 — Parameters of glucose homeostasis among boys and girls according to maternal age at childbirth. Data are 95% confidence intervals for the ratios between estimated marginal means, adjusted for other confounding factors in the multivariate models (including paternal age). Respective p-values are provided in brackets. (DOC) [file pone.0058869.s002.doc]

**Table S2.** Parameters of glucose homeostasis among boys and girls according to maternal age at childbirth. Data are 95% confidence intervals for the ratios between estimated marginal means, adjusted for other confounding factors in the multivariate models (including paternal age). Respective p-values are provided in brackets.

|  | **Maternal age at childbirth** | | |
| --- | --- | --- | --- |
|  | **30–35 vs <30** | **30–35 vs >35** | **<30 vs >35** |
| **Boys** |  |  |  |
| Insulin sensitivity (HOMA-IR) | 0.78 – 1.27  (0.96) | 0.82 – 1.26  (0.90) | 0.75 – 1.39  (0.90) |
| Fasting glucose (mmol/l) | 0.98 – 1.06  (0.29) | 0.96 – 1.03  (0.57) | 0.92 – 1.02  (0.22) |
| Fasting insulin (mU/l) | 0.74 – 1.19  (0.62) | 0.83 – 1.23  (0.91) | 0.80 – 1.42  (0.64) |
| **Girls** |  |  |  |
| Insulin sensitivity (HOMA-IR) | 0.50 – 0.91  **(0.009)** | 0.69 – 1.12  (0.28) | 0.90 – 1.89  (0.16) |
| Fasting glucose (mmol/l) | 0.91 – 0.98  **(0.036)** | 0.95 – 1.03  (0.54) | 0.98 – 1.10  (0.20) |
| Fasting insulin (mU/l) | 0.55 – 0.95  **(0.019)** | 0.72 – 1.12  (0.32) | 0.89 – 1.75  (0.20) |
